# Shengfu Oil Enhances the Healing of Full-Thickness Scalded Skin Accompanying the Differential Regulation of β-Catenin, Dlk1, and COX-2

**DOI:** 10.3389/fphar.2017.00801

**Published:** 2017-11-06

**Authors:** Man-Tang Chen, Yan-Jing Yang, Yu-Sang Li, Xiao-Jun Li, Wei K. Zhang, Jin-Ping Wang, Xu Wang, Gui-Hua Tian, He-Bin Tang

**Affiliations:** ^1^Department of Pharmacology, School of Pharmaceutical Sciences, South-Central University for Nationalities, Wuhan, China; ^2^School of Chemistry and Chemical Engineering, Huazhong University of Science and Technology, Wuhan, China; ^3^Dongzhimen Hospital, Beijing University of Chinese Medicine, Beijing, China; ^4^School of Life Sciences, Beijing University of Chinese Medicine, Beijing, China; ^5^Chinese Evidence Based Medicine Center, West China Hospital, Sichuan University, Chengdu, China

**Keywords:** Shengfu oil, traditional Chinese medicine, wound healing, β-catenin, Dlk1, COX-2

## Abstract

Shengfu oil is a traditional Chinese medicine formula containing 16 ingredients, including Scutellariae radix, Olibanum, and Rehmanniae radix. In this study, we aimed to enhance the wound healing of rabbit full-thickness scalded skin by Shengfu oil and to elucidate its regulatory effects on β-catenin, Dlk1, and COX-2. We found that Shengfu oil exhibited significant anti-inflammatory, analgesic, and antimicrobial activities. The structure of wound tissues in Shengfu oil group was intact, including regenerated cutaneous appendages, indicating better healing capability of Shengfu oil compared to the controls. The protein expression of β-catenin, Dlk1, and COX-2 in wound tissues were investigated by immunohistochemistry staining and were further quantitated with the use of multispectral imaging analysis. The protein expression of β-catenin and Dlk1 in the Shengfu oil group was higher than that in the sesame oil group in early wound repair, accompanied by the lower expression of COX-2; the protein expression of β-catenin decreased in the middle of wound healing; the protein expression of β-catenin and Dlk1 increased at the end of wound healing. These results strongly suggest that Shengfu oil can enhance wound healing by regulating the expression of β-catenin, Dlk1, and COX-2 due to its excellent anti-inflammatory, analgesic, and antimicrobial activities.

## Introduction

Skin can serve as a protective barrier against the environment (Chen et al., 2012). However, physical, chemical, or thermal injuries can easily disrupt the integrity of the skin (Barreto et al., 2014). A delayed, incomplete, or uncoordinated healing process could cause chronic inflammation of the wounds (Agyare et al., 2016). Thus, appropriate wound healing is essential for the restoration of the disrupted anatomical continuity and disturbed functional status of the skin (Singh et al., 2006). The current therapeutic strategies for wound healing, including the treatment of antibiotics, remain to be improved due to the high cost and presence of unwanted side effects (Govindarajan et al., 2004). There is a growing demand for traditional medicine made from plants in developing countries because they are safe and useful for improving healing and reduce the financial burden of treatment (Narayan et al., 2011; Albertyn et al., 2015; Lee et al., 2015). Several plants have been used as traditional medicines to facilitate wound healing (Lodhi et al., 2006; Abdulla et al., 2009; Tan et al., 2014). Even so, more effective and cheaper rapeutic approaches for wound healing are still needed in modern medicine (Barreto et al., 2014).

Shengfu oil, a compounded Chinese medicinal prescription that has effects on saprophytic muscle, anti-inflammatory and analgesic properties, is prepared from 16 traditional drugs, including Scutellariae radix, Olibanum, and Rehmanniae radix, through modern extraction technology (Jia et al., 2013). Our previous work showed that Olibanum, one component of Shengfu oil, exhibited significant anti-inflammatory and analgesic effects (Li X.J. et al., 2016). In 80 first- or second-degree burn patients, clinical observations showed that pain gradually disappeared after treatment with Shengfu oil. Additionally, no wound infection appeared during treatment and no obvious scar was generated after treatment. Moreover, new hair grew eventually. Although the efficacy of each component in Shengfu oil can be explained by the traditional Chinese medicine theory, the detailed characterization of scarless healing by Shengfu oil as an integration are incomplete. Therefore, in this study, we aimed to describe integrally the perfect wound healing effect of Shengfu oil in rabbit.

## Materials and Methods

### Preparation of Shengfu Oil

Briefly, 15 types of Chinese herbal medicines [bark of *Phellodendron amurense* Rupr. (Phellodendri cortex), rhizome of *Rheum officinale* Baill. (Rhei rhizoma), stem of *Astragalus membranaceus* (Fisch.) Bge.var. mongholicus (Bge.) Hsiao (Astragali radix), rhizome of *Coptis chinensis* Franch. (Coptidis rhizoma), root of *Scutellaria baicalensis* Georgi (Scutellariae radix), root of *Angelica dahurica* (Fisch.ex Hoffm.) Benth.et Hook.f. var. *formosana* (Boiss.) Shan et Yuan (Angelicae dahuricae radix), gum resin of *Boswellia carterii* (Olibanum), root of *Sanguisorba officinalis* L. (Sanguisorbae radix), root of *Lithospermum erythrorhizon* Sieb. et Zucc. (Lithospermi radix), resin of *Commiphora myrrha* Eng1 (Myrrh), root of *Rehmannia glutinosa* (Gaert.) Libosch. ex Fisch. et Mey. (Rehmanniae radix), rhizome of *Bletilla striata* (Thunb.) Reichb.f. (Bletillae rhizoma), root of *Cynanchum atratum* Bge. (Cynanchi atrati radix), root of *Angelica sinensis* (Oliv.) Diels (Angelicae sinensis radix), extracts of stem and leaf of *Cinnamomum camphora* (L.) Presl (Camphora); purchased from the Yinpian factory, Guangzhou Medicine Company, Guangzhou, China] were ground into a powder, and soaked in a five times-greater volume of sesame oil under oscillatory mixing conditions for 7 days at room temperature. Thereafter, the mixture was further incubated at 90°C for 2 h, followed by natural cooling to room temperature. Finally, this mixture was filtered through six layers of cotton gauze. The filtered supernatant was saved at 4°C as a Shengfu oil compound for wound healing.

### Evaluation of Shengfu Oil

#### Anti-inflammatory Analysis

To evaluate the anti-inflammatory effect of Shengfu oil, a xylene-induced ear edema mouse model was induced with topical application of 20 μL of a xylene solvent described previously (Hosseinzadeh and Younesi, 2002). Shortly thereafter, 18 male Kunming mice (20–25 g) were randomly divided into three groups of six each, and each mouse was externally treated with a test substance at 0.15 mL/cm^2^ on the surface of the right ear as follows: the Shengfu oil group was treated with Shengfu oil, and DDE group was treated with diclofenac diethylamine emulgel (DDE) as a positive control. The model group was treated with the same amount of a saline solution. Another six male mice without xylene damage (that is control group) were treated with the same amount of a saline solution. The ear thickness was measured using a micrometer at different time points. The left ear remained untreated as a negative control.

#### Analgesic Analysis

To evaluate the analgesic effect of Shengfu oil, a formalin-inflamed mouse hind paw model was established with a formalin intradermal injection (4%, 20 μL) as described previously (Hosseinzadeh and Younesi, 2002). The experimental design was the same as that for the xylene-induced mouse ear edema model. Hind-paw edema and the thermal nociceptive threshold were separately measured using a plethysmometer (Model 7159; UgoBasile, Varese, Italy) and a plantar analgesia meter (Model 37370; UgoBasile, Varese, Italy) at different time points after the formalin injection. The left paw remained untreated as a negative control.

#### Antimicrobial Analysis

The antimicrobial activity of Shengfu oil was analyzed by the standard Kirby-Bauer disk diffusion method (Bauer et al., 1966) against *Staphylococcus aureus* and *Escherichia coli*. The bacterial suspension (10^8^ colony-forming units/mL) was swabbed on beef extract-peptone agar plates using sterile cotton swabs. The sterile disk, which was 5 mm in diameter, was impregnated with the corresponding component. The disks were gently pressed and incubated at 37°C for 20 h. The zone of inhibition in the diameter of each disk was measured in millimeters using a vernier caliper.

### Animals

Male Japanese rabbits (2.2–2.5 kg) were obtained from the experimental animal center of Hubei Province. The care and use of animals for this study were performed according to the Guide for Animal Experimentation, South-Central University for Nationalities. The protocol was approved by the Committee of Research Facilities for Laboratory Animal Sciences, South-Central University for Nationalities, China (Permit Number: 2013-SCUEC-AEC-007).

### Preparation of Scalded Rabbit Model

The animals were anesthetized by pentobarbital sodium (30 mg/kg) and prepared for operation. Water vapor-evoked full-thickness scalded skin with an area of 6 cm^2^ was reproduced on both sides of the back of 36 experimental rabbits after the skin was shaved by an electrical shaver. Then, the scalded rabbits were randomly divided into three groups: sesame oil group (negative group), Shengfu oil group, and mupirocin group (positive group), which were treated with the sesame oil, Shengfu oil, and mupirocin ointment in a dose of 0.15 mL/cm^2^, three times per day, respectively (Jia et al., 2013).

### Macroscopic Observation

The wound façade of rabbits after injury was recorded daily using a single lens reflex camera (Sony α300, Japan) and general changes of the wound surface, mainly including the wound exudation, inflammatory reaction and healing state, were observed at the 1st, 7th, 14th, and 43rd day after the injury.

### Histopathology Staining

Three rabbits in each group were executed by air embolism at the 1st, 7th, 14th, and 43rd day after treatment of wounds with or without Shengfu oil. The wounds with normal tissue were collected and fixed in 10% neutral formalin and then embedded in paraffin. The 4 μm-thick sections were stained with H&E by standard methods and then studied by light microscopy. Furthermore, the histopathological scores were evaluated quantitatively according to the histopathological criteria (Eldad et al., 1998), and the differences between groups at the same time or at different time-points were compared.

### Immunohistochemical Staining and Multispectral Imaging Analyses

To examine the expression of β-catenin, Dlk1, and COX-2, the 4-μm-thick paraffin-embedded skin tissue sections were incubated overnight at 4°C with a rabbit anti-β-catenin antibody (1:500 dilution; Cayman Chemical, Ann Arbor, MI, United States), rabbit anti-Dlk1 antibody (1:300 dilution; Sigma-Aldrich, United States) and rabbit anti-COX-2 (1:400 dilution; Cayman) according to the manufacturer’s instructions [Histofine Simple Stain Rat MAX-PO (MULTI) kit; Nichirei, Tokyo]. Finally, multispectral imaging analyses of all of the slides in each experiment were performed by using an Eclipse Ti microscope (Nikon, Tokyo) with a Nuance Multispectral Imaging System (Cambridge Research and Instrumentation, Inc., Woburn, MA, United States) according to a previously described method (Mansfield, 2010; Li Y.S. et al., 2016). For the quantification in each experiment, five equal-sized fields of each photograph per group were randomly chosen and the average signal strength from the perspective of each positive reaction was measured, and the positive expression rate was calculated (Li et al., 2012).

### Statistical Analysis

All data are presented as the mean ± SEM. Statistical analyses were performed by one- or two-way ANOVA as indicated in the text using Instat software (GraphPad Software, Inc., La Jolla, CA, United States). A *P*-value less than 0.05 was considered statistically significant.

## Results

### Anti-inflammatory Activity of Shengfu Oil

**FIGURE 1:**
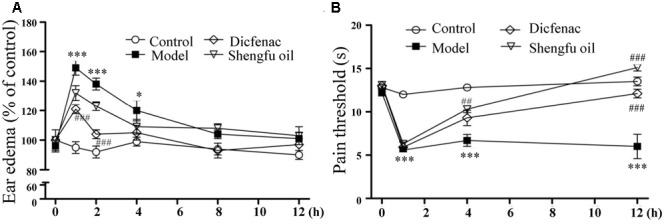
Effects of Shengfu oil treatment on the behavioral responses of mice. **(A)** Return of xylene-induced ear edema at 1, 2, 4, 8, and 12 h after treatment with or without Shengfu oil. **(B)** Return of the formalin-induced rat hind paw thermal nociceptive threshold at1, 2, 4, 8, and 12 h after treatment with or without Shengfu oil. ^∗^*P* < 0.05 and ^∗∗∗^*P* < 0.001 vs. control; ^##^*P* < 0.01 and ^###^*P* < 0.001 vs. model.

As shown in **Figure 1A**, the level of ear edema induced by xylene in the model group increased significantly compared to that in the control group. Compared to the level in the model group (149 ± 5 and 138 ± 4% of the control at 1 and 2 h, respectively), the ear edema level of model mice treated with DDE or Shengfu oil was decreased (121 ± 3 and 104 ± 3% of the control at 1 and 2 h, respectively, for DDE group; 132 ± 5 and 123 ± 3% of the control at 1 and 2 h, respectively, for Shengfu oil group), which showed that Shengfu oil could effectively relieve the inflammatory reaction induced by xylene.

### Analgesic Activity of Shengfu Oil

To evaluate the analgesic effect of Shengfu oil, a formalin-inflamed mouse hind paw model was used. As shown in **Figure 1B**, after the injection of formalin, the thermal pain threshold in the model group decreased significantly compared to that in the control group. Compared to that in the model group, the thermal pain thresholds of mice in the DDE and Shengfu oil groups were all obviously increased, especially in the Shengfu oil group at 12 h (111% of control), which also showed that Shengfu oil could effectively inhibit thermal hyperalgesia induced by formalin and that the efficacy of Shengfu oil treatment was better than DDE.

### Antimicrobial Activity of Shengfu Oil

The antimicrobial activity of Shengfu oil was examined against Gram-positive *Staphylococcus aureus* (ATCC25923) and Gram-negative bacteria *Escherichia coli* (ATCC25922). As shown in **Table 1**, Shengfu oil demonstrated antimicrobial activity against both species.

**Table 1:** The antimicrobial activity of Shengfu oil.

Groups	Test organisms (inhibition zone, mm)	
	*Staphylococcus aureus*	*Escherichia coli*
Control	5.2 ± 0.1 (–)	5.0 ± 0.0 (–)
Low-dose of Shengfu oil	9.2 ± 0.6 (+)	9.0 ± 0.7 (+)
Middle-dose of Shengfu oil	8.4 ± 0.9 (+)	8.7 ± 0.5 (+)
High-dose of Shengfu oil	9.3 ± 0.6 (+)	8.1 ± 0.4 (+)

*+, low activity (6–9 mm).*

### Shengfu Oil Enhanced Wound Healing

**FIGURE 2:**
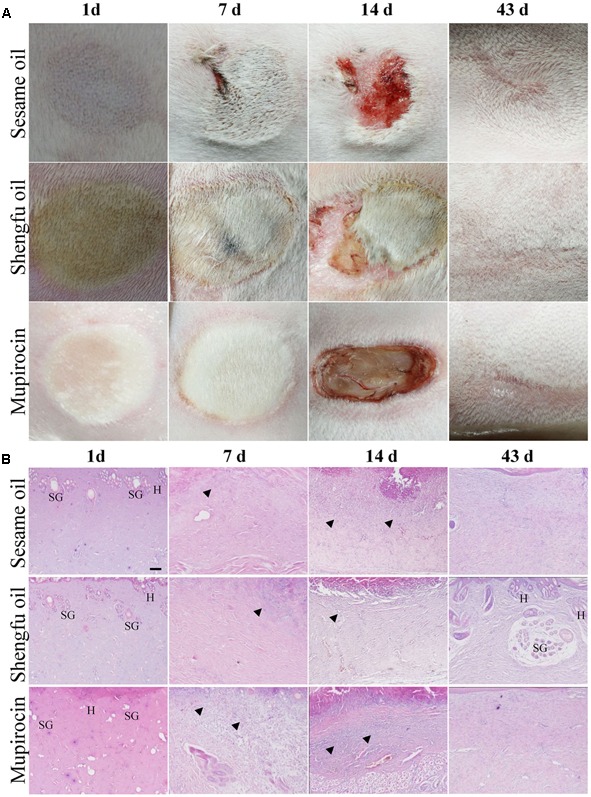
Effects of Shengfu oil treatment on the macroscopic observation and histopathological changes of wounds in scalded rabbits. **(A)** Macroscopic observation of wounds in rabbits at 1, 7, 14, and 43 day(s) after treatment with or without Shengfu oil. Scale bars: 0.5 cm. **(B)** Histopathological changes of wounds in rabbits at 1, 7, 14, and 43 day(s) after treatment with or without Shengfu oil. SG, H and arrows denote sebaceous gland, hair follicle and inflammatory infiltrate, respectively. Scale bars: 100 μm.

As shown in **Figure 2A**, the wounded skin in the sesame oil group and mupirocin group exhibited red swelling, and the margin was slightly hard at the 1st day after being treated with or without Shengfu oil, while the wounded skin in the Shengfu oil group were locally ruddy with inconspicuous swelling and a soft margin. At the 7th day, the wound margin in the sesame oil group was swollen slight bleeding and the wound skins in the mupirocin group were swollen, hard and ruddy, whereas the wounded skin in the Shengfu oil group did not show obvious red swelling and the eschars were smooth. At the 14th day, the wounds in the sesame oil group were smaller. They had scabbed and parts of the scabs fell off. The eschars of the wounded skin in the mupirocin group all fell off, and the tissue under the eschars was exposed. The wounds in the Shengfu oil group were clean, parts of the scabs fell off and new skin grew. At the 43rd day, although the wounds in the sesame oil group and mupirocin group healed, the original wound skins were hard and slightly concave and almost no hair grew. On the contrary, the skin and its surface were smooth and soft for the Shengfu oil group. Moreover, we observed new hair on the original wounded skin.

### Shengfu Oil Enhanced the Regeneration of Cutaneous Appendages

As shown in **Figure 2B**, at the 1st day after treatment with or without Shengfu oil, the skin tissue above the muscle layer in the sesame oil group and mupirocin group necrosed, and part of epibiotic sebaceous glands and hair follicles persisted in the surviving tissue. Moreover, inflammatory infiltration was present in the gap between necrotic tissue and surviving tissue. Compared with the sesame oil group and mupirocin group, no obvious inflammatory infiltrates were present in the gap between necrotic tissue and surviving tissue in the Shengfu oil group. At the 7th day, collagen fibers were disordered and a necrotic cellular structure disappeared in the sesame oil group, while a large number of inflammatory infiltrates were found in the gap between necrotic tissue and surviving tissue in the mupirocin group. Nevertheless, a necrotic cellular structure disappeared and only a few inflammatory infiltrations existed in the Shengfu oil group. At the 14th day, necrotic tissue formed instead of epithelium in scabbed skinin the sesame oil group and mupirocin group. Moreover, no obvious fibroblast proliferation occurred and subcutaneous inflammatory infiltrates were visible. Interestingly, a few epithelia formed and fibroblasts were in alignment. At the 14th day, full epithelia were generated in the sesame oil group and mupirocin group, while no formation of cutaneous appendages was seen and a large wound scar persisted. As expected, in the Shengfu oil group, the wound healed well and neonatal cutaneous appendages, such as the sebaceous gland and hair follicle, formed. Additionally, no obvious scar occurred. The histopathological scores are summarized in **Table 2**.

**Table 2:** Histopathological scores in different groups.

Groups	Score^§^ (mean ± SEM)			
	1st	7th	14th	43rd
Sesame oil group	3.0 ± 0.4	0.9 ± 0.2	1.9 ± 0.1	7.5 ± 0.3
Shengfu oil group	2.6 ± 0.2	3.0 ± 0.0^∗∗∗^	4.6 ± 0.3^∗∗∗^	9.9 ± 0.1^∗∗∗^
Mupirocin group	3.3 ± 0.5	2.6 ± 0.5^∗∗^	2.2 ± 0.5	8.7 ± 0.2^∗^

*^§^Maximal score = 10. ^∗^*P* < 0.05, ^∗∗^*P* < 0.01, and ^∗∗∗^*P* < 0.001 vs. the corresponding sesame oil group.*

### Shengfu Oil Regulated the Protein Expression of β-Catenin, Dlk1, and COX-2

**FIGURE 3:**
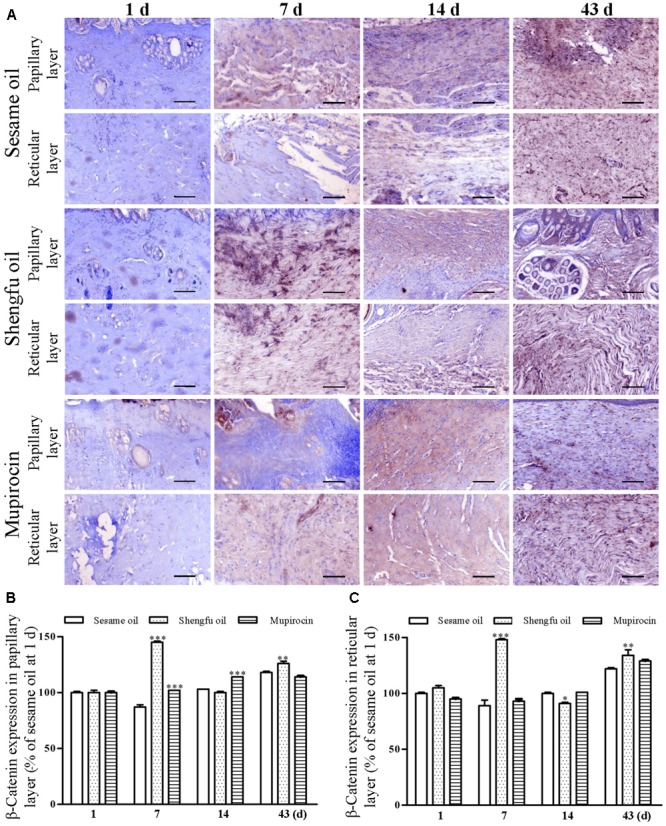
Effect of Shengfu oilon β-catenin expression in scalded local tissues of rabbits. **(A)** Representative photomicrographs of immunohistochemical staining of β-catenin expression in scalded rabbitsat different days after treatment with or without Shengfu oil. Scale bars: 50 μm. Determination of β-catenin expression in the **(B)** papillary layer and **(C)** reticular layer of wounded skin of scalded rabbits using a multispectral analysis. ^∗^*P* < 0.05, ^∗∗^*P* < 0.01, and ^∗∗∗^*P* < 0.001 vs. the corresponding sesame oil group.

**FIGURE 4:**
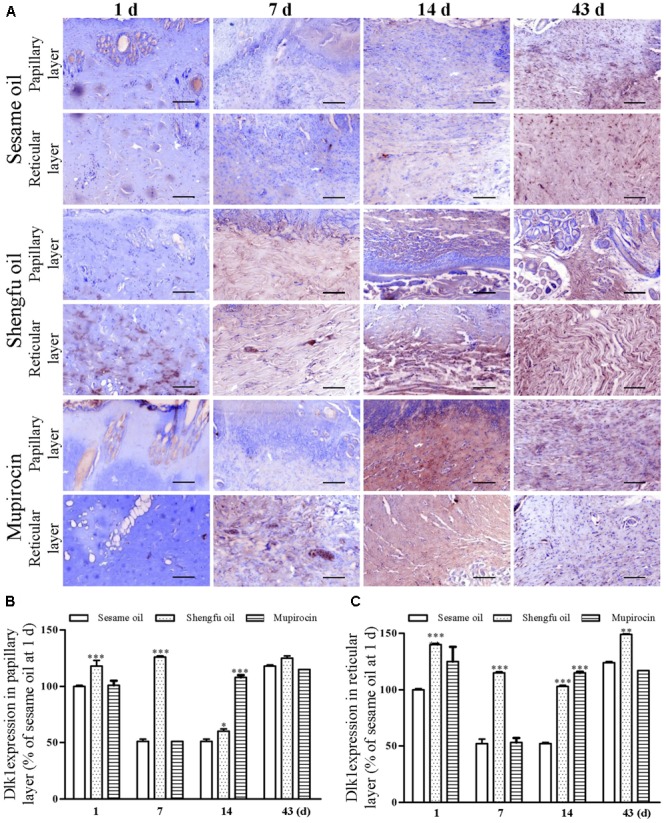
Effect of Shengfu oilon Dlk1 expression in scalded local tissues of rabbits. **(A)** Representative photomicrographs of immunohistochemical staining of Dlk1 expression in scalded rabbits at different days after treatment with or without Shengfu oil. Scale bars: 50 μm. Determination of Dlk1 expression in the **(B)** papillary layer and **(C)** reticular layer of wounded skin of scalded rabbits using a multispectral analysis. ^∗^*P* < 0.05, ^∗∗^*P* < 0.01, and ^∗∗∗^*P* < 0.001 vs. the corresponding sesame oil group.

**FIGURE 5:**
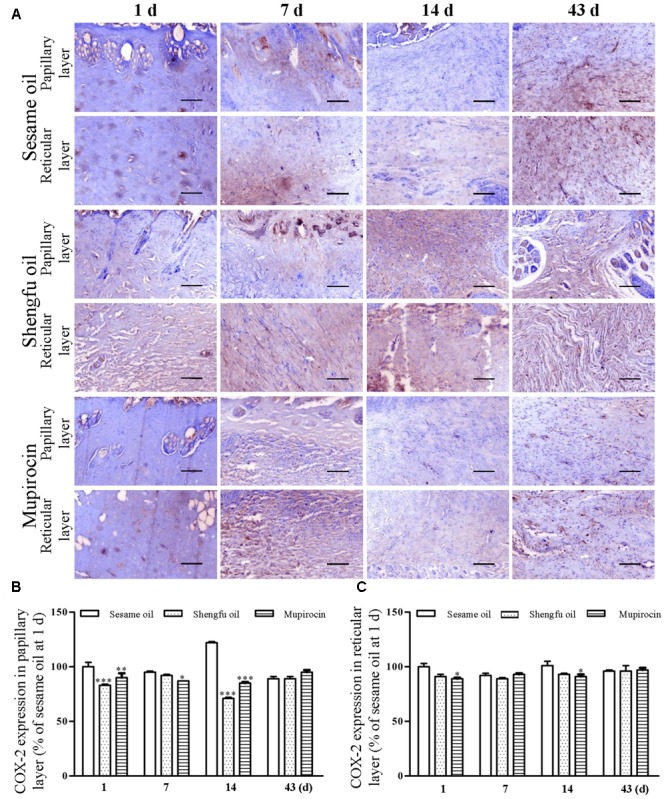
Effect of Shengfu oil on COX-2 expression in scalded local tissues of rabbits. **(A)** Representative photomicrographs of immunohistochemical staining of COX-2 expression in scalded rabbits at different days after treatment with or without Shengfu oil. Scale bars: 50 μm. Determination of COX-2 expression in the **(B)** papillary layer and **(C)** reticular layer of wounded skin of scalded rabbits using a multispectral analysis. ^∗^*P* < 0.05, ^∗∗^*P* < 0.01, and ^∗∗∗^*P* < 0.001 vs. the corresponding sesame oil group.

Immunohistochemical staining showed that the protein expression of β-catenin (papillary layer: 145 ± 1% of sesame oil, *P* < 0.001; reticular layer: 148 ± 1% of sesame oil, *P* < 0.001; **Figure 3**) and Dlk1 (papillary layer: 126 ± 1% of sesame oil, *P* < 0.001; reticular layer: 115 ± 1% of sesame oil, *P* < 0.001; **Figure 4**) in the Shengfu oil group were higher than those in the sesame oil group (β-catenin: 87 ± 2% of sesame oil in papillary layer, 89 ± 5% of sesame oil in reticular layer; Dlk1: 51 ± 2% of sesame oil in papillary layer, 52 ± 1% of sesame oil in reticular layer; papillary layer: 95 ± 1% of sesame oil; reticular layer: 92 ± 2% of sesame oil) in early wound repair, accompanied by the lower expression of COX-2 (papillary layer: 92 ± 1% of sesame oil; reticular layer: 89 ± 1% of sesame oil; **Figure 5**); the protein expression of β-catenin (papillary layer: 100 ± 1% of sesame oil; reticular layer: 91 ± 1% of sesame oil; **Figure 3**) fell in the middle of wound repair; the protein expression of β-catenin (papillary layer: 126 ± 2% of sesame oil, *P* < 0.01; reticular layer: 134 ± 5% of sesame oil, *P* < 0.05; **Figure 3**) and Dlk1 (papillary layer: 125 ± 2% of sesame oil; reticular layer: 149 ± 1% of sesame oil; **Figure 4**) increased at the end of wound repair.

## Discussion

In this study, we confirmed that Shengfu oil exhibited significant anti-inflammatory, analgesic and antimicrobial activities and that Shengfu oil could enhance wound healing by regulating the expression of β-catenin, Dlk1, and COX-2.

Wound healing is a succession of complicated biochemical and cellular events that aims to restore the structural and functional integrity of wounded tissue (Fronza et al., 2014). It is characterized by a sequence of in-dependent and/or overlapping events (Diegelmann and Evans, 2004; Chan et al., 2008). These events can be broadly categorized into three stages: the inflammatory phase, proliferative phase and remodeling phase (Hayashi et al., 2009).

Wound healing is influenced by many factors that mainly rely on the proliferation and migration of fibroblasts. In late wound healing, the inhibition of cell apoptosis induces the over proliferation of fibroblasts, which leads to the formation of a scar. Several proteins, such as β-catenin, Dlk1, and COX-2, are closely associated with wound healing. Recent research showed that the overexpression of β-catenin can induce the differentiation of epidermal stem cells toward hair (Li et al., 2013), which will promote the regeneration of hair follicles. Nevertheless, the sustained activation of the Wnt/β-catenin signaling pathway can induce the increased proliferation of adult fibroblasts and the expression of intracellular transforming growth factor β3, and the abnormal down-regulation of hyaluronan synthases 2 and 3 nucleic acid expression (Carre et al., 2010), which are adverse factors for wound healing. Fibroblasts expressing Dlk1 in the late embryonic development are located in the skin mesh layer, which is mainly responsible in wound healing. Meanwhile, fibroblasts located in the skin’s papillary layer are essential for the generation of hair follicles (Driskell et al., 2013). In the process of wound healing, inflammatory reactions will up-regulate the production of COX-2 and the secretion of prostaglandins, activate the vascular endothelial growth factor (Enzerink et al., 2010), promote the neogenesis of wound vessels and accelerate the blood supply, which contribute to rapid wound healing (de Oliveira et al., 2014). However, the overexpression of COX-2 leads to severe inflammation, which adversely affects wound healing.

Skin wounds cause serious damage to people’s physical and mental health and economic benefits. Our team developed a strategy for the external use of Shengfu oil in the treatment of wounds, and the characteristic chromatogram of Shengfu oil was established by GC-MS (Supplementary Figure 1). The present study showed that Shengfu oil exhibited excellent anti-inflammatory, analgesic activity (**Figure 1**) and good antibacterial activity (**Table 1**). Besides, the mainly component of Shengfu oil is sesame oil, which can keep the wound in a moist environment to enhance the wound healing (Xiao et al., 2014). Based on our results, it is reasonable to conclude that Shengfu oil can effectively treat wounds. As expected, Shengfu oil not only enhanced wound healing of rabbit full-thickness scalded skin but also enhanced the regeneration of cutaneous appendages (**Figure 2**). To elucidate its regulatory mechanism, we further quantitatively analyzed changes in the expression of β-catenin, Dlk1, and COX-2 during Shengfu oil-enhanced wound healing of rabbit full-thickness scalded skin. After treatment with Shengfu oil, the expression of β-catenin in the papillary layer and reticular layer of wounded skin increased remarkably in early wound repair. The activation of β-catenin initiated the differentiation and migration of the epithelium, promoted epithelial coverage of the wound surface and accelerated wound healing. Meanwhile, the protein expression of Dlk1 was maintained at a high level, indicating the induction of Dlk1 expression, which could also promote wound healing. Moreover, the expression of COX-2 in both the papillary layer and reticular layer was inhibited, which decrease wound inflammation. In the middle of wound repair, the protein expression of β-catenin was down-regulated in favor of the normal apoptosis of pathological fibroblasts, thus hindering the appearance of scars. At the end of wound repair, the strong expression of β-catenin and Dlk1 facilitated wound remodeling. Moreover, the neonatal epithelial layer and granulation tissue differentiated and transformed and the collagenous fiber was rearranged to achieve good wound healing.

## Conclusion

We demonstrate here that Shengfu oil can enhance scarless wound healing by regulating the expression of β-catenin, Dlk1 and COX-2, exerting anti-inflammatory, analgesic, and antimicrobial effects. It may serve as a promising potent therapeutic agent for the treatment of full-thickness scalded skin.

## Author Contributions

Conceived and designed the experiments: H-BT. Performed the experiments: M-TC, Y-JY, and H-BT. Analyzed the data: H-BT, M-TC, Y-SL, X-JL, WZ, J-PW, and XW. Contributed reagents/materials/analysis tools: H-BT and Y-SL. Wrote the paper: H-BT, M-TC, Y-SL, X-JL, WZ, and G-HT.

## Conflict of Interest Statement

The authors declare that the research was conducted in the absence of any commercial or financial relationships that could be construed as a potential conflict of interest.
