# Supplementary material for: Shengfu Oil Enhances the Healing of Full-Thickness Scalded Skin Accompanying the Differential Regulation of β-Catenin, Dlk1, and COX-2
Source: Front Pharmacol. 2017 Nov 6;8:801. doi: 10.3389/fphar.2017.00801 (PMC5681749; doi:10.3389/fphar.2017.00801)
Supplement: Supplementary file 1 [file Presentation_1.pdf]

Supplementary Material

Shengfu oil enhances the healing of full-thickness scalded skin accompanying the differential regulation of β-catenin, Dlk1, and COX-2

Man-Tang Chen[1, 2,¶], Yan-Jing Yang[1, ¶], Yu-Sang Li[1], Xiao-Jun Li[1], Wei Kevin Zhang[1], Jin-Ping Wang[2], Xu Wang[3], Gui-Hua Tian[2,4, *], He-Bin Tang[1,*]

*** Correspondence:** Gui-Hua Tian: rosetgh@163.com;

He-Bin Tang: hbtang2006@mail.scuec.edu.cn

1 GC-MS analysis of Shengfu oil

The chemical components of Shengfu oil were detected by GC-MS on a Thermo Scientific TRACE 1300 GC Ultra system equipped with a TR-35MS capillary column (30 m×0.25 mm×0.25 μm) coupled to an ISQ system in electron ionization mode. The initial oven temperature was 50°C. Samples were maintained at 50 °C for 1 min, and the temperature was increased at a rate of 5°C/min to 200°C, where it was held for 2 min. The injector and transfer line temperatures were both 290°C.The injection volume was 1 μL. Ions were generated by a 70 eV electron beam at an ionization current of 50 μA and an ion source temperature of 250°C. The mass spectra were recorded in full scan mode (m/z 50-500) for qualitative analysis. The characteristic chromatogram of Shengfu oil has been established by GC-MS and can be acted as the quality standard to evaluate and confirm the product. Hexanal, terpinen, terpinolene, linalool and (1S)-(+)-camphorquinone may be the main components of Shengfu oil, and most of which have been reported to be related to anti-inflammatory, analgesic, and antimicrobial activities [1-9]. It should be pointed out that the exact chemical components corresponding to peaks in the spectrogram will be unriddled in future.

[1] Li, X. J., Yang, Y. J., Li, Y. S., Zhang, W. K., and Tang, H. B. (2016). α-pinene, linalool, and 1-octanol contribute to the topical anti-inflammatory and analgesic activities of frankincense by inhibiting COX-2. Journal of Ethnopharmacology, 179, 22.

[2] Sajid, M., Khan, M. R., Shah, S. A., Majid, M., Ismail, H., & Maryam, S., et al. (2017). Investigations on anti-inflammatory and analgesic activities of alnus nitida, spach (endl). stem bark in sprague dawley rats. Journal of Ethnopharmacology, 198, 407-416.

[3] Tashiro, S., Ran, Y., Ishikawa, S., Sakurai, T., Kajiya, K., & Kanmura, Y., et al. (2016). Odour-induced analgesia mediated by hypothalamic orexin neurons in mice. Scientific Reports, 6, 37129.
[4] Aprotosoaie, A. C., Hăncianu, M., Costache, I., & Miron, A. (2014). Linalool: a review on a key odorant molecule with valuable biological properties. Flavour & Fragrance Journal, 29(4), 193-219.
[5] Mátis, G., Kulcsár, A., Petrilla, J., Talapka, P., & Neogrády, Z. (2016). Porcine hepatocyte-kupffer cell co-culture as an in vitro model for testing the efficacy of anti-inflammatory substances. Journal of Animal Physiology & Animal Nutrition, 101(2), 201.
[6] Yadav, E., Kumar, S., Mahant, S., Khatkar, S., & Rao, R. (2016). Tea tree oil: a promising essential oil. Journal of Essential Oil Research, 1-13.
[7] Caldefiechézet, F., Fusillier, C., Jarde, T., Laroye, H., Damez, M., & Vasson, M. P., et al. (2006). Potential anti-inflammatory effects of melaleuca alternifolia essential oil on human peripheral blood leukocytes. Phytotherapy Research Ptr, 20(5), 364-70.
[8] Kazemi, M. (2014). Chemical composition, antimicrobial, antioxidant and anti-inflammatory activity of l. essential oil. Journal of Essential Oil Bearing Plants, 17(5), 1002-1011.
[9] Cabral, C., Poças, J., Gonçalves, M. J., Cavaleiro, C., Cruz, M. T., & Salgueiro, L. (2015). Ridolfia segetum, (l.) moris (apiaceae) from portugal: a source of safe antioxidant and anti-inflammatory essential oil. Industrial Crops & Products, 65, 56-61.

2 Supplementary Figure

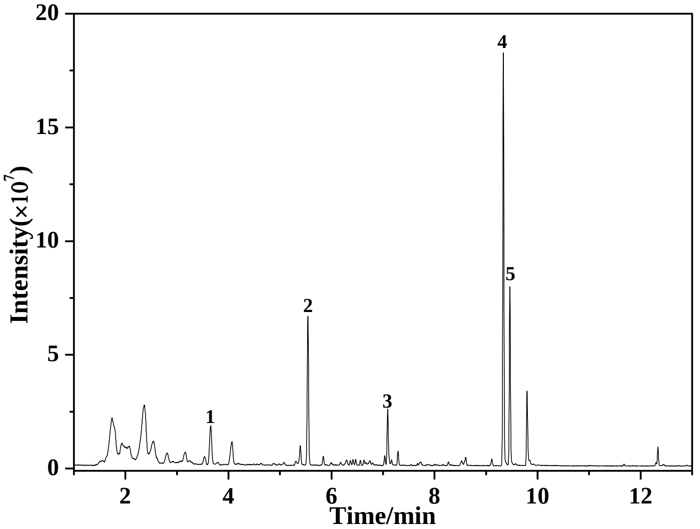

Supplementary Figure 1.GC-MS analysis of the components of Shengfu oil. The numbers in the graph from 1 to 5 may denote hexanal, terpinen, terpinolene, linalool and (1S)-(+)-camphorquinone, respectively.
